# Fidelity of peripheral blood for monitoring genomics and tumor immune‐microenvironment in myelodysplastic syndromes

**DOI:** 10.1002/jha2.112

**Published:** 2020-10-05

**Authors:** Sung‐Eun Lee, Feng Wang, Abel Trujillo‐Ocampo, Wilfredo Ruiz‐Vasquez, Hyun‐Woo Cho, Koichi Takahashi, Jeffrey J. Molldrem, Andrew Futreal, Guillermo Garcia‐Manero, Jin S. Im

**Affiliations:** ^1^ Department of Stem Cell Transplantation and Cellular Therapy, Division of Cancer Medicine The University of Texas M.D. Anderson Cancer Center Houston Texas USA; ^2^ Department of Hematology, Seoul St. Mary's Hospital, College of Medicine The Catholic University of Korea Seoul South Korea; ^3^ Department of Genomic Medicine, Division of Cancer Medicine The University of Texas M.D. Anderson Cancer Center Houston Texas USA; ^4^ Department of Leukemia, Division of Cancer Medicine The University of Texas M.D. Anderson Cancer Center Houston Texas USA; ^5^ Department of Hematopoietic Biology and Malignancy, Division of Cancer Medicine The University of Texas M.D. Anderson Cancer Center Houston Texas USA

**Keywords:** bone marrow aspirate, genomics, myelodysplastic syndrome, peripheral blood, tumor immune‐microenvironment

## Abstract

The aim of this study is to investigate whether the peripheral blood (PB) can serve as a surrogate immune‐microenvironment to bone marrow for genetic and immune monitoring in myelodysplastic syndrome (MDS). We compared the composition of T cell subsets and somatic mutation burden in 36 pairs of PB and matching bone marrow aspirate (BMA) using multi‐parameter flow cytometry and NGS‐based targeted sequencing analysis, respectively. Our immune‐subset and NGS‐based mutation analysis of BMA showed significant concordance with those of PB in MDS. Therefore, PB can provide easily accessible tumor immune‐microenvironment for monitoring in the immune and genetic landscapes for MDS patients.

Myelodysplastic syndrome (MDS) is a disorder of hematopoiesis characterized by cytopenias, dysplasia, and a propensity to transform into acute myeloid leukemia (AML). The clinical and pathological features of this hematologic malignancy are driven by complex genetic mutations [1]. Especially, mutations in key epigenetic regulators in MDS can silence several immune‐related genes in tumor and tumor microenvironment and may contribute to the immune dysregulation leading to ineffective antitumor immunity [2]. Therefore, further investigation is in need to understand how genetic alterations affect tumor immune‐surveillance and immune‐landscape in tumor microenvironment of MDS.

Hypomethylating agents (HMAs) such as 5‐azacytidine and 5‐aza‐2′ deoxycytidine are standard treatment for patients with high risk MDS, but the resistance to HMAs occurs in most MDS patients leading to a dismal outcome [3]. Recently, immune checkpoint proteins were reportedly upregulated on leukemia blasts and immune cells from MDS patients during HMA treatment, and thought to be one of acquired immune‐mechanism of resistance to HMA [4]. This finding led to multiple clinical trials evaluating HMAs in combination with immune checkpoint blockade for MDS patients. Close monitoring of immune and genetic landscapes in tumor microenvironment during immunotherapy will tremendously aid to decipher critical determinants for early clinical responses in MDS patients during combination immunotherapy.

The presence of tumor‐infiltrating T cells (TIL) in tumor or high mutation burden in tumor immune‐microenvironment are such examples of predictive immune‐biomarkers associated with clinical responses to immunotherapy in solid tumor [5, 6], however, most investigations of biomarkers in solid tumor have been performed in tumor microenvironment, not peripheral blood. This is because peripheral blood in solid tumor may not fully represent the complexity of infiltrating immune‐subsets and tumor heterogeneity in tumor immune‐microenvironment, although neo‐antigen‐specific T cells similar to TIL can be isolated from peripheral blood [7] and circulating tumor cells from peripheral blood may provide some insight on the biology of cancer metastasis [8].

Unlike solid tumors, malignant clones co‐exist with other immune subsets in both peripheral blood and bone marrow in hematologic malignancies. Accordingly, peripheral blood may serve as an alternative tumor immune‐microenvironment for investigating genetic and immune biomarkers predictive or prognostic to immunotherapy in hematologic malignancies, especially myelodysplastic syndrome given the low burden of disease. Next generation sequencing (NGS)‐based mutation evaluation has been a valuable tool in prognosis prediction and treatment decision for MDS patients [9, 10, 11]. Bone marrow aspirates have been the choice of tumor microenvironment to assess somatic mutation analysis in MDS patients but recent advances in NGS technology raises a question on how accurately mutation landscapes from peripheral blood represent those in bone marrow [12]. More recently, Takahashi et al. reported that clonal hematopoiesis in patients with therapy‐related myeloid neoplasm could be detected from the peripheral blood samples at the time of primary cancer diagnosis [13], suggesting that NSG‐based mutation analysis from peripheral blood may be a reasonable approach to represent the genetic landscape in MDS patients. Here, we compared the composition of T cell compartments and somatic mutation burden in peripheral blood and matching bone marrow aspirates, to ascertain the fidelity of the peripheral blood as a monitoring site for tumor and immune response from MDS patients.

Thirty‐six pairs of peripheral blood and matching bone marrow aspirates were obtained from 23 MDS patients at the time of diagnosis or during treatment, and processed within 24 h from the collection, and characteristics of patients were described in Table S1. Freshly prepared PBMCs and BMMCs (34 pairs) were subjected to multi‐parameter flow cytometry (MFC) analysis for the following markers: CD3, CD4, CD8α, HLA‐DR, CD45RA, CD62L, CD161, CD25, CD127, CD16, CD56, and fixable viable stain reagents to exclude dead cells. After fixation with 2% paraformaldehyde, cells were acquired using LSR Fortessa Cell Analyzer (BD Bioscience, Franklin Lakes, NJ), and subsequent analysis was performed using Flowjo version 10.3 (Tree Star, Ashland, OR) (Figure S1). NGS‐based targeted sequencing analysis of 295 myeloid‐associated genes was performed to an average depth of 443 on DNAs extracted from 24 pairs of PB or bone marrow aspirates (BMAs), and high‐confidence mutations were selected according to previously published methodology [13]. Paired Student *t*‐test was used to compare differences between two groups, Pearson's correlation coefficient was calculated to assess the strength of linear association between two variables. Lastly, Bland‐Altman plot was employed to show the differences from the average values, bias (mean of differences from average), as well as 95% limits of agreement. All statistical analysis was performed using GraphPad Prism 8.0. *P*‐value < .05 was considered as statistically significance. All patients provided written, informed consents, and research was conducted in accordance with the Declaration of Helsinki and The University of Texas MD Anderson Cancer Center Institutional Review Board guidelines.

First, we compared each T‐cell subset between PB and BMA by paired Student *t*‐test (Figure 1). While there were no statistical significant differences between PB and BMA in CD4^+^ T cells, CD8α^+^ T cells, CD4^−^CD8α^−^ T cells, Tregs, CD161^+^CD4^+^ T cells, and CD161^+^CD8α^+^ T cells (Figure 1A,B), BMA contained statistically higher frequencies of HLA‐DR^+^CD4^+^ T cells (median [95% CI] PB vs BM, hereafter; 1.36 [1.13, 2.09] vs 2.38 [1.48, 3.52], *P* = .0223) and CD8α^+^ T cells (1.11 [0.94, 1.53] vs 2.35 [1.47, 2.74], *P* = .0123) than PB (Figure 1B). This is likely because of higher disease burden in bone marrow than peripheral blood in MDS. Nonetheless, all major T cell subsets from BM including activated T cells showed statistically significant concordant values to those from peripheral bloods (Figure S2). Among memory subtypes of T cells such as naïve (CD45RA^+^CD62L^+^), CD45RA^+^ effector memory (TEMRA, CD45RA^+^CD62L^−^), effector memory (EM, CD45RA^−^ CD62L^−^), and central memory (CD45RA^−^ CD62L^+^) T cells, BM contained lower frequencies of CD45RA^+^ effector memory (TEMRA) CD4^+^ T cells (2.24 [0.55, 8.70] vs 1.95 [0.90, 3.98], *P* = .0259) and higher frequencies of effector memory (EM) CD8α^+^ T cells (25.9 [15.3, 36.0] vs 36.1 [26.2, 38.3], *P* = .0129) than PB, although there was no significant difference in other memory subsets of T cells in both CD4^+^ and CD8α^+^ T cells (Figure 1C,D). Again, all memory T cell subsets from BM were highly concordant to those from PB (Figure S2). Lastly, Bland‐Altman plot analysis demonstrated that the lines of agreements were included within 1 standard deviation from mean of difference (bias) and >95% of values were within 95% limits of agreements in most immune‐subsets analyzed (Figure S3 and Table S2). In summary, out results suggest that PB can be an alternative tumor immune‐microenvironment for monitoring the immune‐landscape during treatment.

**FIGURE 1:**
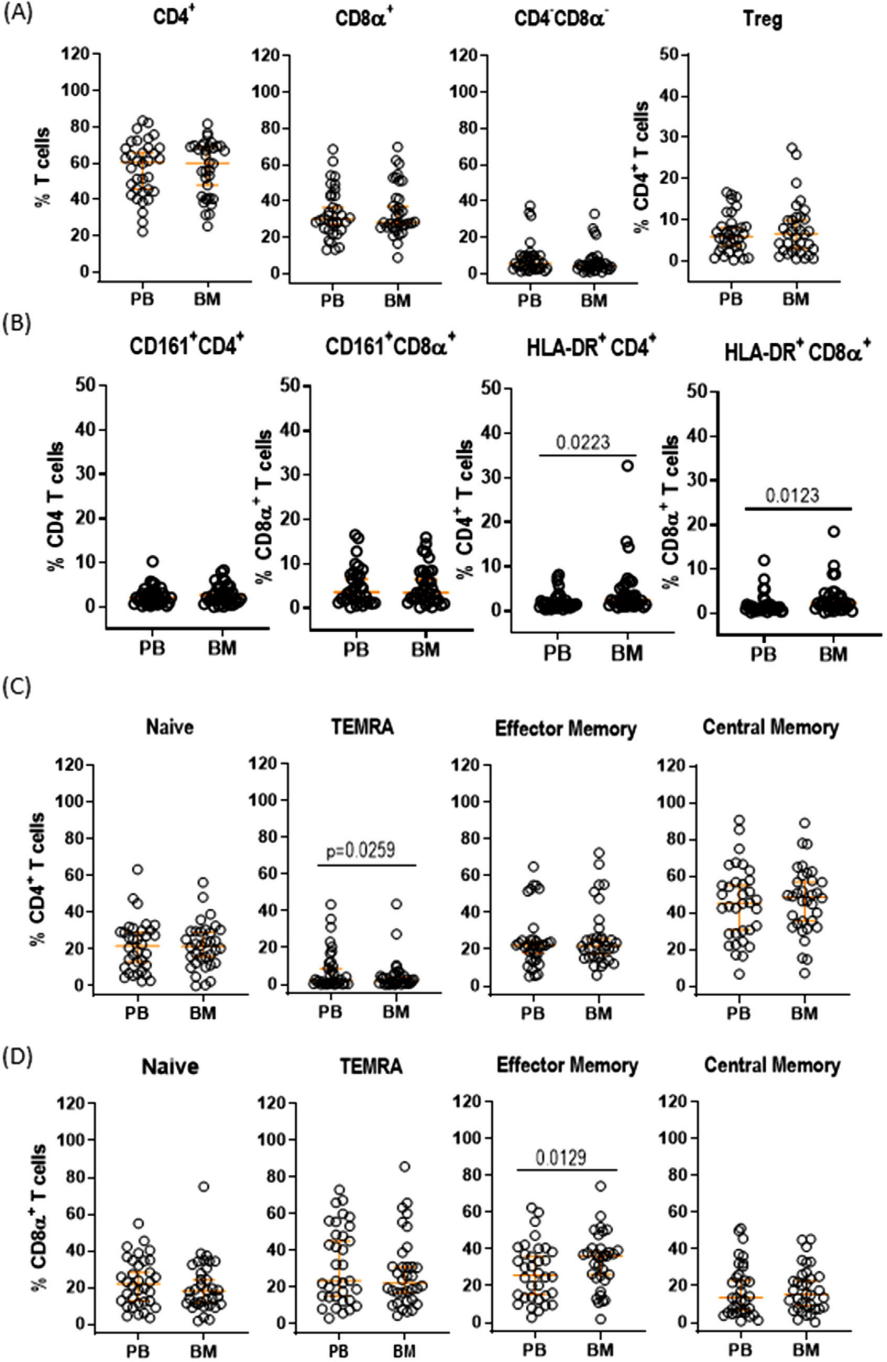
**Comparison of T‐cell subsets between bone marrow aspirates (BMAs) and paired peripheral blood (PB)**. The frequency of CD4^+^, CD8α^+^, and CD4^−^CD8α^−^ T cells, and Treg in BMA and PB (A). CD161^+^CD4^+^ and CD161^+^CD8α^+^ T cells, and activated T cells expressing HLA‐DR in BMA and PB (B). The frequency of naïve (CD45RA^+^CD62L^+^), CD45RA^+^ effector memory (TEMRA, CD45RA^+^CD62L^−^), effector memory (CD45RA^−^CD62L^−^), and central memory (CD45RA^−^CD62L^+^) CD4^+^ T cells in BMA and PB (C). The frequency of naïve (CD45RA^+^CD62L^+^), CD45RA^+^ effector memory (TEMRA, CD45RA^+^CD62L^−^), effector memory (CD45RA^−^CD62L^−^), and central memory (CD45RA^−^CD62L^+^) CD4^+^ T cells in BMA and CD8^+^ T cells (D). A symbol represents a value from single donor. The data were presented as the median with 95% CI. The paired sample *t*‐test was used to assess differences between groups. *P*‐value was presented only when it was <.05

Next, we performed targeted gene sequencing of BMA and matching PB to investigate whether there are discrepancies in quality and quantity of non‐synonymous somatic mutations (Figure 2). Not only were there no significant differences present in the number of somatic mutations between BMA and PB shown by paired *t*‐test (Figure 2A, left and middle), but also the number of mutations were highly concordant between BMA and PB (Pearson's *r* = 0.9862, *P* < .001; Figure 2A, middle). As expected, the sum of Variant Allelic Frequencies (VAF) of all detectable mutations from BM was significantly higher than those from PB (Figure 2B, left), but they were highly correlated to those from PB (Pearson's *r* = 0.9571, *P* < .001; Figure 2B, middle). Further, BM contained significantly higher mutation burden in both individual drivers and all mutations (Figure 2C,D, left). VAF of individual driver mutations of PB significantly correlated to those of BM (Pearson's *r* = 0.8374, *P* < .001; Figure 2C, middle), and there was again a significant correlation of VAF of all individual mutations between PB and BMA (Pearson's *r* = 0.8499, *P* < .001; Figure 2D middle). The Bland‐Altman plot analysis confirmed that bias toward higher mutation burden was present in BM compared to PB (Figure 2B‐D, right). Last and most importantly, all driver mutations were present in both PB and BM, and NGS‐mutation analysis failed to detect only one of 42 mutations from PB. Therefore, PB can serve as surrogate tumor microenvironment for monitoring the mutation landscape via NGS‐based mutation analysis in MDS patients.

**FIGURE 2:**
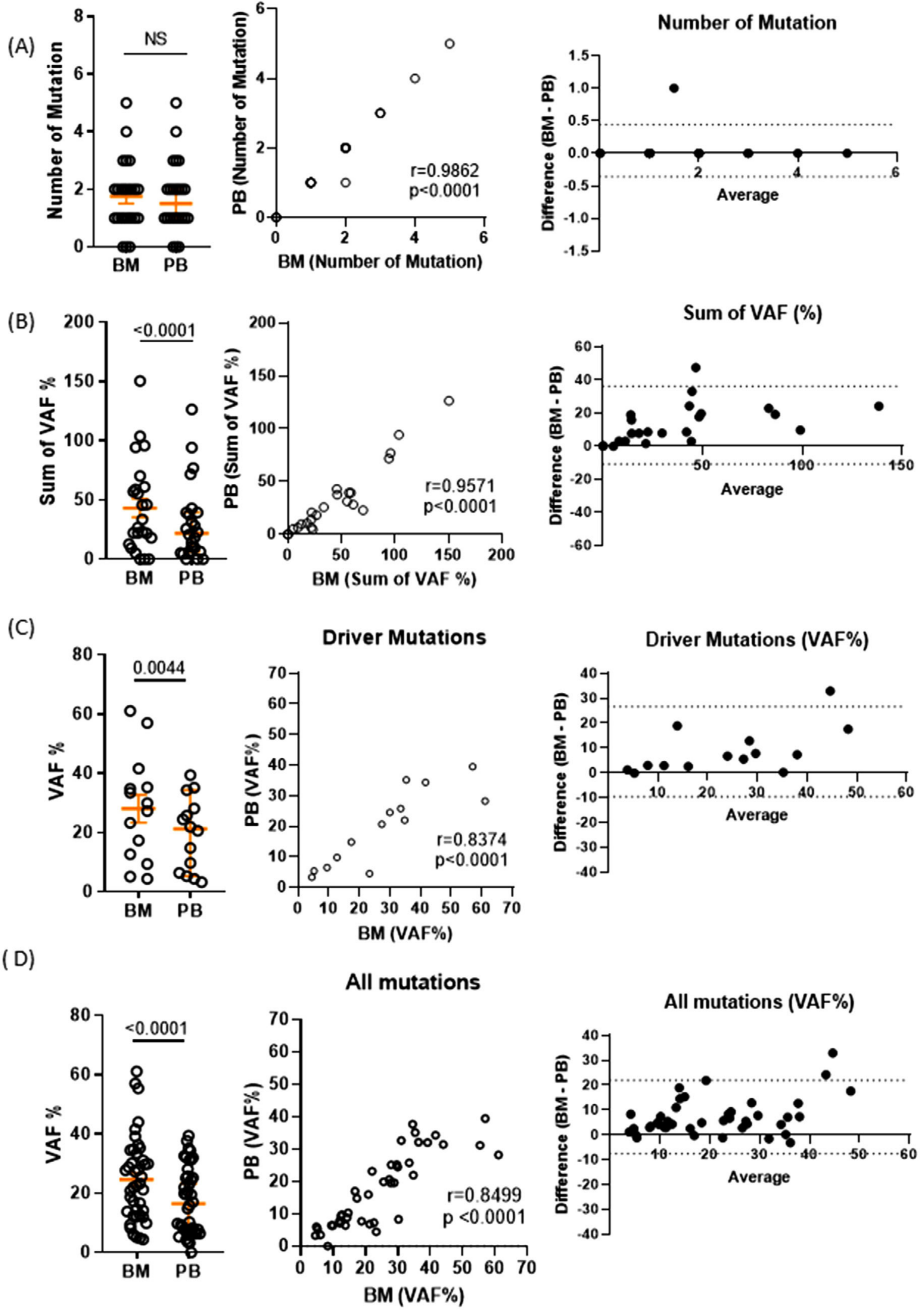
**Mutation landscape of peripheral bloods and bone marrow is highly concordant**. Comparison and correlation analyses of mutation numbers detected between BMA and PB (A), sum of VAF between BMA and PB (B). Comparison and correlation analysis of VAF of individual driver mutations and all individual mutations between PB and BMA (C and D). A symbol represents a value from single donor. The paired sample *t*‐test was used to assess differences between groups (left), Pearson's correlation coefficient was used to assess correlation between paird values from two groups (middle), and the differences and average were shown by Bland‐Altman plot (right). *P*‐value was presented only when it was <.05

Daver et al. published the results from phase 2 study of azacitidine and nivolumab in relapsed/refractory acute myeloid leukemia [14]. In their exploratory immune subset analysis, patients who achieved a response with azacitidine and nivolumab had higher CD3^+^, effector CD4^+^ T, and CD8^+^ T cells in the pretreatment BMA, and higher CD3^+^ T cells in pretreatment PB. In addition, the increase in frequencies of CTLA‐4^+^ CD4^+^ or CD8^+^ T cells in the BM after treatment was associated with non‐responders. This work suggests that analysis of baseline and changes of immune subsets in BM of patients with AML can aid in selecting potential responders for immunotherapy and monitoring subsequent responses during treatment. However, whether peripheral blood can be used for immune‐monitoring in AML patients remains unclear and it may not be feasible as AML tends to have higher disease burdenin bone marrow than peripheral blood.

Different from AML, our immune‐subset and NGS‐based mutation analysis of BM showed significant concordance with those of peripheral bloods in MDS. Therefore, peripheral blood in especially elderly MDS patients will provide easily accessible tumor immune‐microenvironment for monitoring of dynamic changes in the immune and genetic landscapes during combination immunotherapy with hypomethylator agents [15]. However, it is important to note that peripheral blood may not replicate BM stromal cell microenvironment [16]. MDS is a clinically dynamic and evolving disease, where clonal selection and subsequent immune‐escape may contribute to disease progression. Thus, close monitoring of the immune and genetic landscapes in PB would benefit and facilitate developing personalized immunotherapy for MDS patients.

## FUNDING INFORMATION

This work was funded through the Catholic Medical Center Research Foundation (S.L.), the MD Anderson Cancer Center Leukemia SPORE 2P50CA100632 (J.I.), Khalifa Bin Zayed Al Nahyan Foundation (J.I.), Emerson Collective Cancer Research foundation (J.I.), MDACC Institutional Start‐up Fund (J.I.), Welch Foundation (A.F), Lydia Hill foundation (A.F.), Anna Darko foundation (A.F.), MDACC MDS/AML Moonshot (G.G.M), and NCI Cancer Center Support Grant P30CA16672 (Flow Cytometry and Cellular Imaging Facility).

## AUTHOR CONTRIBUTIONS

S.L, H.C, W.R., A.T and., J.I. performed the experiments; S.L, J.I, K.T., and F.W. interpreted the results; and S.L, F.W, H.C., W.R., A.T., K.T., J.M, G.G, A.F, and J.I. wrote the manuscript.

## CONFLICT OF INTEREST

Authors declare there is no competing financial interest in relation to the work described.

## Supporting information

Supplementary Fig 1. Gating strategies

Supplementary Fig 2. Correlation analyses of T‐cell subsets between BMA and paired PB. A symbol represents a value from single donor. Pearson's correlation coefficient was used to assess correlation between paired values from two groups, and P value was presented only when it was less than 0·05.

Supplementary Fig 3. Difference and average of T‐cell subsets between BMA and paired PB. A symbol represents a value from single donor. The differences were presented as the value of PB minus that of BM.

Supplementary Table I. Characteristics of patients.
Supplementary Table II. The Bland‐Altman results of T‐cell subsets between BMA and paired PB
Supplementary Table III. The Bland‐Altman results of mutation number, sum of VAF, VAF of individual driver mutations and all individual mutations between BMA and paired PB

## Data Availability

The data of the current study are available from the corresponding author on a reasonable request.
