# Supplementary figures and images for: Fidelity of peripheral blood for monitoring genomics and tumor immune‐microenvironment in myelodysplastic syndromes
Source: EJHaem. 2020 Oct 5;1(2):552–7. doi: 10.1002/jha2.112 (PMC9175915; doi:10.1002/jha2.112)

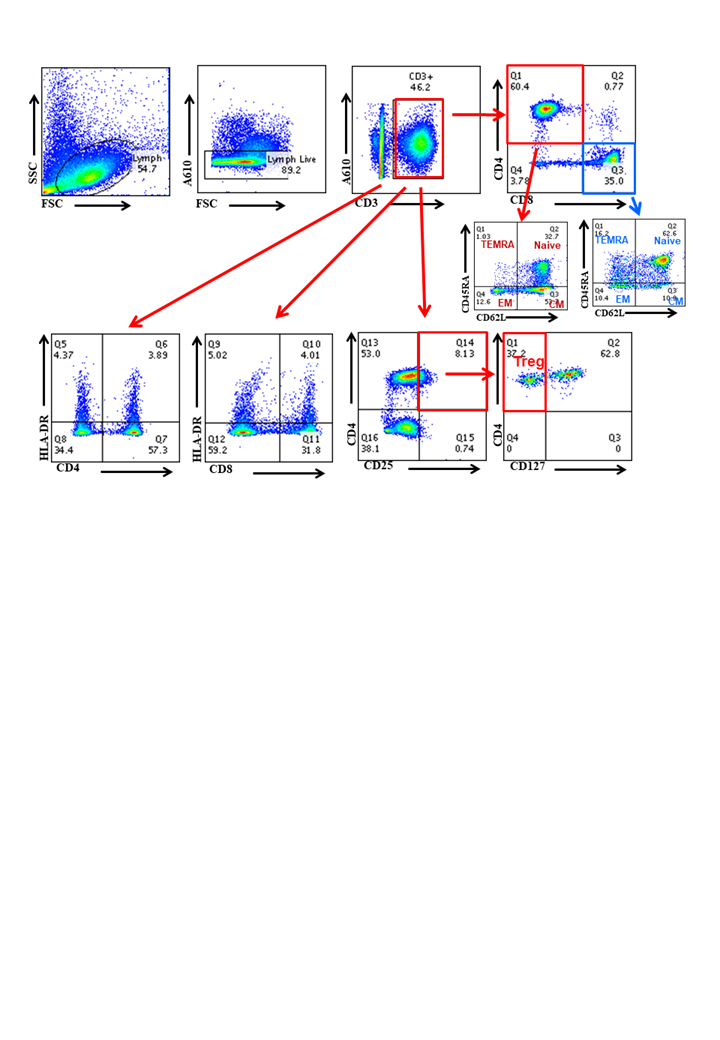

Supplement: Supplementary file 1 — Supplementary Fig 1. Gating strategies [file JHA2-1-552-s003.tif]

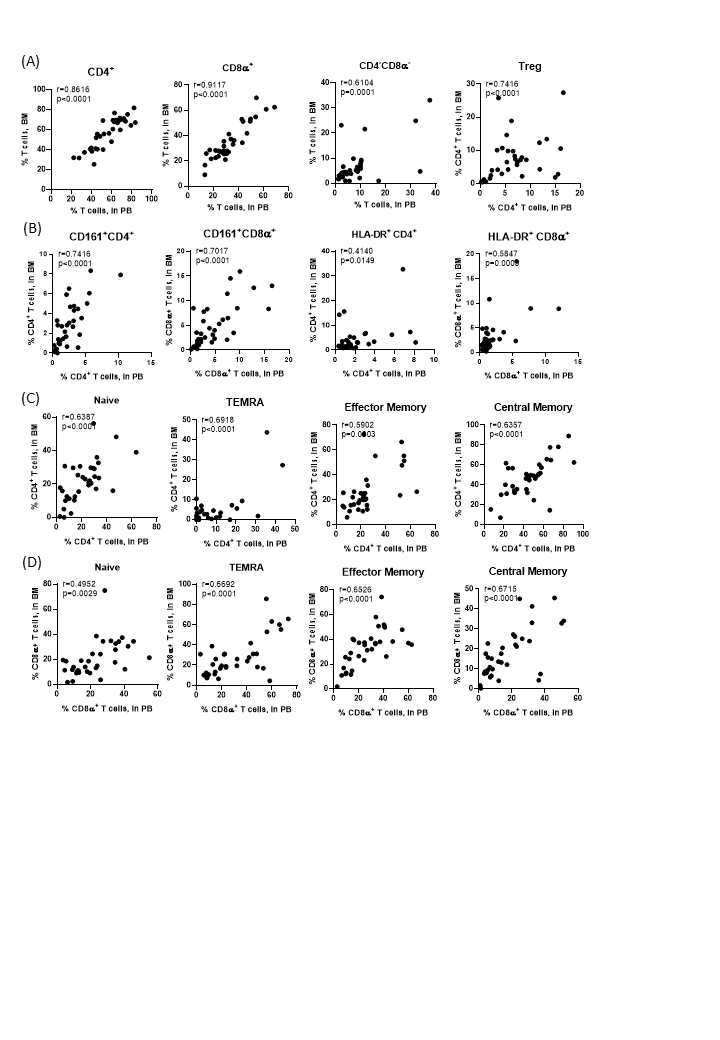

Supplement: Supplementary file 2 — Supplementary Fig 2. Correlation analyses of T‐cell subsets between BMA and paired PB. A symbol represents a value from single donor. Pearson's correlation coefficient was used to assess correlation between paired values from two groups, and P value was presented only when it was less than 0·05. [file JHA2-1-552-s002.tif]

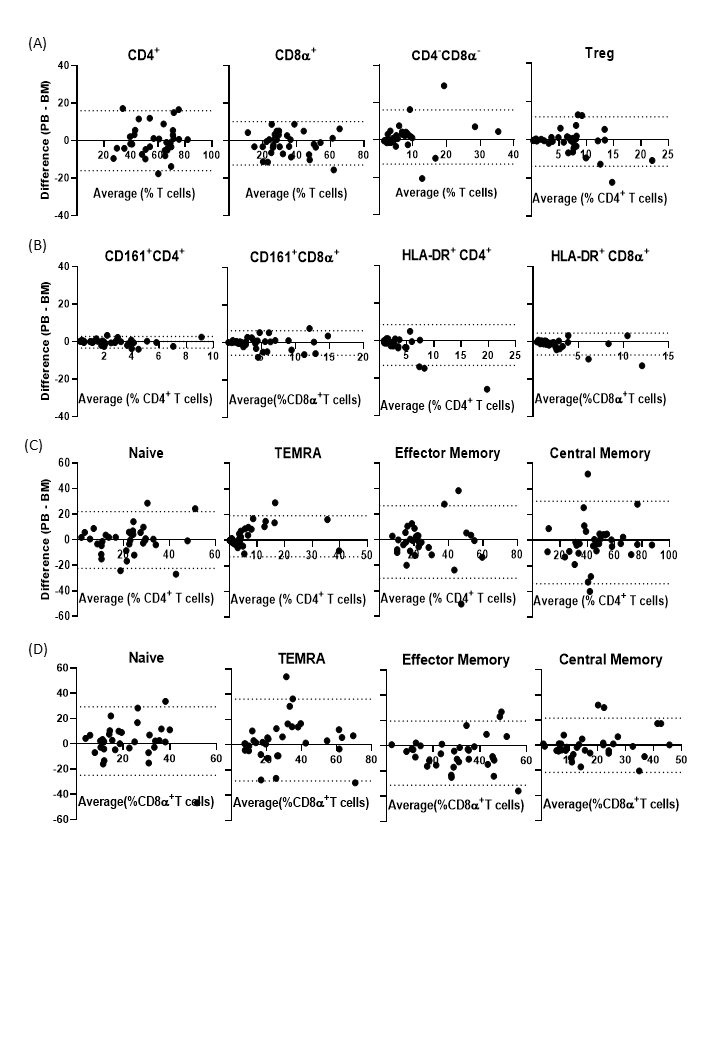

Supplement: Supplementary file 3 — Supplementary Fig 3. Difference and average of T‐cell subsets between BMA and paired PB. A symbol represents a value from single donor. The differences were presented as the value of PB minus that of BM. [file JHA2-1-552-s004.tif]
