# Supplementary material for: Fidelity of peripheral blood for monitoring genomics and tumor immune‐microenvironment in myelodysplastic syndromes
Source: EJHaem. 2020 Oct 5;1(2):552–7. doi: 10.1002/jha2.112 (PMC9175915; doi:10.1002/jha2.112)
Supplement: Supplementary file 4 — Supplementary Table I. Characteristics of patients. Supplementary Table II. The Bland‐Altman results of T‐cell subsets between BMA and paired PB Supplementary Table III. The Bland‐Altman results of mutation number, sum of VAF, VAF of individual driver mutations and all individual mutations between BMA and paired PB [file JHA2-1-552-s001.docx]

**Supplementary Table I. Characteristics of patients**

| Variables | No. (%) |
| --- | --- |
| No. of patient | 23 |
| No. of paired sample | 36 pair |
| Used for MFC | 34 pair |
| Used for NGS-based mutation | 24 pair |
| Age, years, median (range) | 67 (22 – 79) |
| Gender |  |
| Male | 15 (65) |
| Female | 8 (35) |
| WHO classification at diagnosis |  |
| EB-1 | 9 (39) |
| EB-2 | 7 (30) |
| CMML | 2 (9) |
| Others | 5 (22) |
| IPSS-R cytogenetic risk |  |
| Very good/Good | 4 (17) |
| Intermediate | 9 (39) |
| Poor/Very poor | 10 (44) |
| Monosomal karyotype |  |
| No | 14 (61) |
| Yes | 9 (39) |
| IPSS-R risk |  |
| Very low/Low | 4 (17) |
| Intermediated | 6 (26) |
| High/Very high  (NA=2) | 11 (48) |
| Treatment |  |
| ICI + HMA | 14 (61) |
| HMA | 8 (35) |
| None | 1 (4) |
| **Laboratory data matched with each sample** |  |
| WBC count, x 10^9^/L, median (range) | 1·60 (0·5-7·6) |
| Absolute neutrophil count, x 10^9^/L, median (range) | 0·42 (0·0-3·4) |
| Lymphocyte count, x 10^9^/L, median (range) | 0·86 (0·28-3·8) |
| Marrow blast, median (range) | 2 (0-22) |

CMML, chronic myelomonocytic leukemia; EB-1, excess blast-1; EB-2, excess blast-2; HMA, hypomethylating agent; ICI, immune checkpoint inhibitor; IPSS-R, Revised International Prognostic Scoring System; MFC, multiparameter flow cytometry; NA, not available

**Supplementary Table II. The Bland-Altman results of T-cell subsets between BMA and paired PB**

|  | Difference | | 95% Limits of Agreement | |
| --- | --- | --- | --- | --- |
|  | Mean | Standard deviation | From | To |
| CD4^+^ , % T cells | -0.082 | 8.154 | -16.060 | 15.900 |
| CD8α^+^, % T cells | -1.466 | 5.915 | -13.060 | 10.130 |
| CD4^-^CD8α^-^, % T cells | 1.606 | 7.406 | -12.910 | 16.120 |
| Tregs, , % CD4^+^ T cells | -0.871 | 6.572 | -13.750 | 12.010 |
| CD161^+^CD4^+^, % CD4^+^ T cells | -0.406 | 1.570 | -3.483 | 2.671 |
| CD161^+^CD8α^+^, % CD8α^+^ T cells | -0.221 | 3.329 | -6.746 | 6.305 |
| HLA-DR^+^CD4^+^, % CD4^+^ T cells | -2.278 | 5.539 | -13.130 | 8.579 |
| HLA-DR^+^CD8^+^, % CD8α^+^ T cells | -1.348 | 2.969 | -7.167 | 4.471 |
| Naïve, % CD4^+^ T cells | -0.086 | 11.340 | -22.310 | 22.140 |
| TEMRA, % CD4^+^ T cells | 3.271 | 8.173 | -12.750 | 19.290 |
| Effector Memory, % CD4^+^ T cells | -1.608 | 14.490 | -30.020 | 26.800 |
| Central Memory, % CD4^+^ T cells | -1.579 | 16.410 | -33.750 | 30.590 |
| Naïve, % CD8α^+^ T cells | 2.168 | 13.810 | -24.910 | 29.240 |
| TEMRA, % CD8α^+^ T cells | 3.722 | 16.540 | -28.700 | 36.140 |
| Effector Memory, % CD8α^+^ T cells | -5.850 | 12.970 | -31.280 | 19.580 |
| Central Memory, % CD8α^+^ T cells | -0.039 | 11.000 | -21.600 | 21.520 |

**Supplementary Table III. The Bland-Altman results of mutation number, sum of VAF, VAF of individual driver mutations and all individual mutations between BMA and paired PB**

|  | Difference | | 95% Limits of Agreement | |
| --- | --- | --- | --- | --- |
|  | Mean | Standard deviation | From | To |
| Number of mutation | 0.042 | 0.204 | -0.358 | 0.442 |
| Sum of VAF, % | 12.560 | 12.010 | -10.970 | 36.090 |
| VAF of individual driver mutations, % | 8.503 | 9.264 | -9.655 | 26.660 |
| VAF of all individual mutations, % | 7.175 | 7.513 | -7.551 | 21.900 |
